# Cost-Effectiveness of Aspirin Adjuvant Therapy in Early Stage Colorectal Cancer in Older Patients

**DOI:** 10.1371/journal.pone.0107866

**Published:** 2014-09-24

**Authors:** Swee Sung Soon, Whay-Kuang Chia, Mun-ling Sarah Chan, Gwo Fuang Ho, Xiao Jian, Yan Hong Deng, Chuen-Seng Tan, Atul Sharma, Eva Segelov, Shaesta Mehta, Raghib Ali, Han-Chong Toh, Hwee-Lin Wee

**Affiliations:** 1 Department of Pharmacy, National University of Singapore, Singapore, Singapore; 2 Department of Medical Oncology, National Cancer Centre Singapore, Singapore, Singapore; 3 Department of Radiation Oncology, University of Malaya Medical Centre, Kuala Lumpur, Malaysia; 4 Department of Medical Oncology, Sixth Affiliated Hospital, Sun Yat-sen University, Guangzhou, China; 5 Saw Swee Hock School of Public Health, National University of Singapore, Singapore, Singapore; 6 Department of Oncology, All India Institute of Medical Sciences, New Delhi, India; 7 National Health and Medical Research Council Clinical Trials Centre, University of Sydney, Sydney, Australia; 8 Department of Digestive Diseases and Nutrition, Tata Memorial Hospital, Mumbai, India; 9 Nuffield Department of Population Health, University of Oxford, Oxford, United Kingdom; National Institute for Public Health and the Environment, Netherlands

## Abstract

**Background & Aims:**

Recent observational studies showed that post-operative aspirin use reduces cancer relapse and death in the earliest stages of colorectal cancer. We sought to evaluate the cost-effectiveness of aspirin as an adjuvant therapy in Stage I and II colorectal cancer patients aged 65 years and older.

**Methods:**

Two five-state Markov models were constructed separately for Stage I and II colorectal cancer using TreeAge Pro 2014. Two hypothetical cohorts of 10,000 individuals at a starting age of 65 years and with colorectal cancer in remission were put through the models separately. Cost-effectiveness of aspirin was evaluated against no treatment (Stage I and II) and capecitabine (Stage II) over a 20-year period from the United States societal perspective. Extensive one-way sensitivity analyses and multivariable Probabilistic Sensitivity Analyses (PSA) were performed.

**Results:**

In the base case analyses, aspirin was cheaper and more effective compared to other comparators in both stages. Sensitivity analyses showed that no treatment and capecitabine (Stage II only) can be cost-effective alternatives if the utility of taking aspirin is below 0.909, aspirin’s annual fatal adverse event probability exceeds 0.57%, aspirin’s relative risk of disease progression is 0.997 or more, or when capecitabine’s relative risk of disease progression is less than 0.228. Probabilistic Sensitivity Analyses (PSA) further showed that aspirin could be cost-effective 50% to 80% of the time when the willingness-to-pay threshold was varied from USD20,000 to USD100,000.

**Conclusion:**

Even with a modest treatment benefit, aspirin is likely to be cost-effective in Stage I and II colorectal cancer, thus suggesting a potential unique role in secondary prevention in this group of patients.

## Introduction

Colorectal cancer (CRC) is the third most common cancer worldwide with more than 1.2 million new cases diagnosed annually [1]. More than half of the patients diagnosed with CRC die from the disease and it is the second leading cause of overall cancer deaths in the United States [2]. Over the past decade, coincident with a rapid rise in CRC incidence rates in Asia [3], there is a dramatic increase in the proportions of CRC patients diagnosed with early stage disease [4]–[6].

Adjuvant chemotherapy has been shown to reduce the risk of recurrence and improve overall survival (OS) in patients with Stage III CRC. Chemotherapy with 5-fluorouracil reduces the relative risk (RR) of cancer recurrence by approximately 30%, and absolute risk by approximately 15% [7]. However, adjuvant chemotherapy has a much more limited role in earlier stages of CRC (Stage I and II) where its benefit is modest at best, and limited to tumors with high risk features in patients under 70 years [8], [9].

Most recently, data from a series of observational studies have strongly supported a beneficial role of aspirin use after CRC diagnosis, with a halving of disease-specific mortality rates [10]. In these analyses, aspirin’s effectiveness was not restricted to Stage III tumors, but extended to Stage I and II disease. Large randomized adjuvant studies are now underway in Asia (NCT00565708) and Europe (NTR3370) to confirm the benefit of aspirin in CRC patients.

Since aspirin is cheap, easy to administer, and has a good risk-benefit profile relative to chemotherapy, we hypothesize that aspirin might represent a cost-effective strategy for the adjuvant treatment of Stage I and II CRC where the risk of cancer recurrence is low. Such patients are currently not routinely offered adjuvant chemotherapy and are followed-up with observation alone. As the number needed to treat (NNT) to prevent one CRC recurrence or death will be much larger for Stage I and II CRC than for Stage III disease, global cost-effectiveness will be an important consideration for advocating treatment in low relapse-risk cancers.

To date, although there have been several cost-effectiveness analyses of aspirin in the primary prevention of CRC [11]–[13], no studies have been undertaken to evaluate the cost-effectiveness of aspirin in the adjuvant or secondary cancer prevention setting. Given the ever escalating costs of cancer care and constraints in health resources globally, a cost-effectiveness analysis of adjuvant aspirin in the context of treatment of cancer, in particular low-risk cancer, is both timely and important. The primary objective of this study is to determine the cost-effectiveness of aspirin as adjuvant therapy for Stages I and II CRC in the United States (U.S.) population. The U.S. was chosen as the population under study due to the relative availability of data for model input. The study model focused solely on sporadic CRC as it is the most common and relevant type of CRC [14].

## Methods

### Model Structure

**Figure 1:**
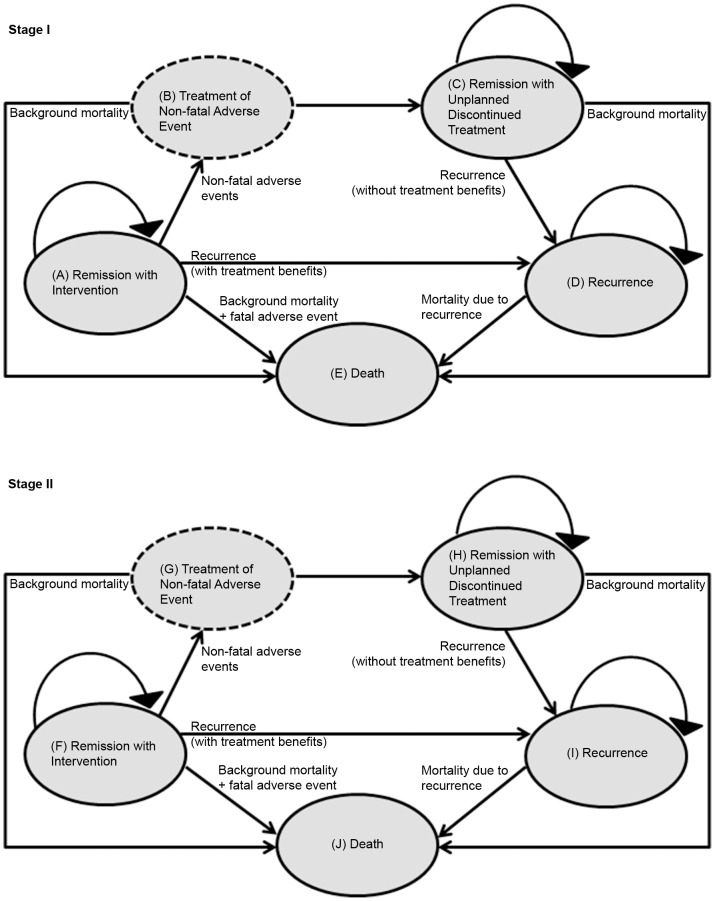
Markov Models for Stage I and II Colorectal Cancer. Patients enter the model at the ‘Remission with Intervention’ state for both stages. For the ‘no treatment’ arm of both stages, fatal and non-fatal adverse events are taken to be zero. ‘Treatment of Non-fatal Adverse Event’ state is modeled as a temporary state where patients remain in that state for only one cycle.

Based on literature review and clinicians’ input, two separate Markov cohort models for Stage I and Stage II CRC respectively were constructed using TreeAge Pro 2014 (TreeAge Software, Inc., Williamstown, MA). Although the health states were identical, the state-specific transition probabilities, efficacy and utility estimates differed according to cancer stage. The five health states were: ‘Remission with Intervention’, ‘Treatment of Non-fatal Adverse Event’, ‘Remission with Unplanned Discontinued Treatment’, ‘Recurrence’, and ‘Death’ (Figure 1). In Stage I, the treatment options were aspirin or no treatment; and in Stage II, aspirin, chemotherapy or no treatment. The chemotherapy regime selected was the standard protocol of capecitabine, an oral pro-drug of 5-fluorouracil. Capecitabine was used as a comparator in Stage II disease as it is an oral agent, has better side effects profile than 5-fluorouracil [15], and is commonly used in the treatment of Stage II CRC patients with high-risk tumor features.

A hypothetical cohort of 10,000 individuals at a starting age of 65 years was simulated in each model that had a cycle length of one year and ran over 20 years. The entry age of 65 years was selected since the median age at diagnosis for Stage I and II colon and rectum cancers ranged from 66 to 73 [16]. Based on the average life expectancy of 19.1 years at 65 years of age in 2010 [17], a time horizon of 20 years was chosen. All subjects entered the model at the ‘Remission with Intervention’ health state, received the intervention specified, and then progressed through the model in annual cycles. The U.S. societal perspective was adopted for the analysis and published data on cost inputs from public databases and cost studies were utilized. Health outcomes were measured in terms of incremental cost per quality-adjusted life year (QALY), and incremental cost per life year gained (LYG).

### Model Assumptions

The model assumed a uniform treatment benefit effect and fatality risk across the various regions of the ascending, transverse, descending and sigmoid colon and rectum, and all treatment effects were assumed to be immediate. Disease-free survival ratios and cancer-specific survival ratios for capecitabine and aspirin respectively were used for the imputation of treatment benefit in the model [9], [18]. Patients who experienced Grade 3 or 4 adverse events (AE) from aspirin or capecitabine were assumed to discontinue their use. In addition, the model assumed that no more than one AE could occur within each cycle. The risks of treatment-related side effects were assumed to cease immediately after completion of adjuvant treatment (five years for aspirin and six months for capecitabine), and after treatment was prematurely terminated due to serious AEs. After five years, the risk of death from other causes was thought to be equal to that of the general population of the same age. Bleeding risk was estimated from cardiovascular aspirin studies and assumed to be equal in patients with resected CRC. Bleeding risk from aspirin was assumed to be uniform across the exposure period and beneficial effects of intervention were assumed to apply during the five-year aspirin regimen. Similarly, for capecitabine, the beneficial effects were assumed to apply for the first five years of the simulation.

All patients were assumed to be treatment naïve at the beginning of treatment. For simplicity, all recurrences were deemed incurable although it is recognized that 6% from Stages I, II and III could be expected to have surgically curable recurrences [19]. Hence, the transition from ‘Recurrence’ back to either of the ‘Remission’ states was not permissible in our model. Aspirin’s cardiovascular benefit and chemoprevention effects on other cancers were not included in this model.

### Transition Probabilities

Transition probabilities refer to the likelihood of an event happening in a given time period and differ from rates which are instantaneous. The transition matrices (File S2) show the probability of transition from states in the rows to states in the columns.

### Model Validation

To ensure face validity, the model structures and assumptions were developed in consultation with medical oncologists. The no treatment arm was then validated using data derived from the Surveillance, Epidemiology, and End Results (SEER) Program SEER*Stat Database (version 8.1.2) to simulate natural history of early stage CRC. The details of the validation using SEER data can be found in File S1.

### Treatment Effects

To model for the beneficial effects of aspirin and capecitabine, the relative risk of disease recurrence on aspirin or chemotherapy versus no treatment was applied to the transition probabilities associated with disease progression. Treatment effect of aspirin was specific to standard oral 325 mg aspirin tablet daily[18]. Treatment effect of oral capecitabine was assumed to be equivalent to that of the intravenous administration of 5-fluorouracil using the Mayo Clinic regimen [15]. Estimates of the beneficial effects were taken from the QUASAR study as their study population, with 91% of enrolled patients having Stage II disease, was the most similar to our hypothetical cohort [9].

For aspirin, age-related fatal (hemorrhagic death) and non-fatal (major gastrointestinal bleeding and intracranial bleeding) AEs were used [20]–[22]. For capecitabine-related side-effects, both non-fatal AE (Grade 3 or 4 hand-and-foot syndrome and diarrhea) and fatal AE were included in the model [15], [23].

The QALY and LYG were summed across all model cycles. Incremental effectiveness was estimated as the difference across treatment arms in terms of QALY or LYG.

### Utilities

The respective stage-specific mean utility scores for staying in remission for Stage I and II CRC were estimated from stage-specific utilities elicited from CRC patients [24]. For the recurrence state, mean utility scores for Stage IV were applied. A utility of 0.999 (i.e. disutility of 0.001) was applied to the period aspirin was taken [25]. This represents the diminution of quality of life due to inconvenience of taking a daily pill [25], [26].

### Cost Inputs

As the societal perspective was adopted, direct medical costs, indirect medical costs and non-medical costs were considered (Table 1). These included costs of drugs (aspirin and capecitabine), surveillance (physician visit, blood tests, serum carcinoembryonic antigen (CEA) level test, computerized tomography and colonoscopy), medical care (cost of care for metastatic CRC), adverse events and indirect costs (patient’s time). Costs relating to surveillance used non-facility rates in the Medicare Physician Fee Schedule from the Centers for Medicare & Medicaid Services [27], while those relating to adverse events were extracted from the Healthcare Costs and Utilization Project (HCUP) based on charges billed for clinically meaningful categories developed by the Agency for Healthcare Research and Quality [28]. All costs were inflated to 2013 (November) U.S. dollars using the Medical Care Consumer Price Index [29] and were discounted at 5% in the base case, while outcomes was discounted at a lower 3% to take into account the increase in future value of health effects [30]. All costs and outcomes were taken to be incurred at the end of the year.

**Table 1:** Cost Inputs for Stage I & II Colorectal Cancer Patients.

Item	Frequency	Unit cost in US Dollars (Year)	Unit cost in 2013 US Dollars (Range)^a^	Derivations (Source)	Stage I				Stage II			
					Remission	Treatment of non-fatal AE	Remission (discontinued)	Recurrence	Remission	Treatment of non-fatal AE	Remission (discontinued)	Recurrence
**Drug Cost**												
Aspirin	Daily 325 mg standard tablet (for 5 years)	18 per year (2000)	30 per year (24–36)	Referenced [13]	X				X			
Capecitabine	1,250 mg/m2 two times a day, 21 days per cycle, 8 cycles in 6 months (for 6 months)	7,263 per month (2006)	9,261 per month (7,409–11,114)	Referenced [31]					X			
**Surveillance**	**Cost** b											
Physician visit	3 monthlyc (Years 1–2)	143 per visit (2013)	143 per visit (114–172)	Referenced [27]	X	X	X	X	X	X	X	X
Blood test	3 monthly (Year 1)	11 per administration (2013)	11 per administration (8.6–12.8)	Referenced [45]	X	X	X	X	X	X	X	X
Serum CEA level test		26 per administration (2013)	26 per administration (20.9–31.3)		X	X	X	X	X	X	X	X
Computerized tomography (thorax, abdomen, pelvis)	Yearly	410 per administration (2013)	410 per administration (328–492)	Referenced [27]	X	X	X	X	X	X	X	X
Colonoscopy	Yearly for Years 1 and 4, then every 5 yearly	745 per administration (2011)	785 per administration (628–942)	Referenced [46]				X				X
**Indirect**	**Cost** d **^,^** e											
Patient’s time when in remission	11 hours per year	24 per hour (2013)	255 per year (204–306)	Time estimates for remission stage were taken from the continuing phase using 0.88 hours per month for office visits	X	X	X		X	X	X	
Patient’s time when in recurrence	432 hours per year	24 per hour (2013)	10,437 per year (8,350–12,524)	time estimates for recurrence stage were taken from the terminal phase using 432.17 hours per year for the terminal phase [32]. Median U.S. wage rate in November 2013 at an hourly value of USD24.15 [47]				X				X
**Medical**	**Care Cost** f											
Cost of care for metastatic CRC	Yearly	117,576 per year (2008)	138,453 per year (110,762–166,144)	Metastatic CRC- related costs amounted to USD9,798 per month [35]				X				X
**Adverse**	**Events Cost**											
Aspirin – Non- fatal major gastrointestinal bleeding	Age-specific, from 0.0023–0.0058 g	21,700 per episode (2005)	Weighted average cost per non-fatal-episode: Age- specific, from 32,656–37,301 (26,125–44,761)	Referenced [20]–[22], [48], [49]		X				X		
Aspirin – Non-fatal intracranial bleeding	Age-specific, from 0.0013–0.0052 g	32,400 per episode (2005)				X				X		
Aspirin – Fatal major gastrointestinal bleeding	Age-specific, from 0.000072–0.00312 g	21,700 per episode (2005)	Weighted average cost per fatal episode: Age-specific, from 41,056–42,442 (32,845–50,931)	For deaths resulting from fatal aspirin adverse events only								
Aspirin – Fatal major intracranial bleeding	Age-specific, from 0.00069–0.00276 g	32,400 per episode (2005)		Referenced [20]–[22], [48], [49]								
Capecitabine – Non-fatal side effectsh	0.0291	674 per month (2006)	5,157 per year (4,125–6,188)	Derivation based on a 6-month regimen. [31], [50]						X		
Capecitabine – Fatal side effectsh	0.003	50,248 per episode (2011)	53,820 per episode (43,056–64,583)	For deaths resulting from fatal capecitabine adverse events only. Weighted average for pneumonia, septicemia, organ failure was taken. For organ failure, mean charges relating to renal, respiratory, hepatic, cardiac systems [51] were first averaged before using the mean for the calculation of the weighted average. [23], [52]								

a The Medical Consumer Price Indexes were 1.64 (November 2013/2000), 1.33 (November 2013/2005), 1.28 (November 2013/2006), 1.22 (November 2013/2007), 1.07 (November 2013/2011). [29] All cost range adopted was ±20%, except for costs exceeding USD100,000, cost range of ±50% was applied. Point estimates used in base case analyses and ranges used in sensitivity analyses.

b Since the societal perspective is adopted, the non-facility costs were used for cost estimates extracted from the Centers for Medicare & Medicaid Services (CMS) so as to capture the resources utilized in the provision of the service.

c For capecitabine, during the six-month chemotherapy regimen, the frequency of physician visit is every three weeks.

d Applied only for the first 3 years of the cohort simulation using the full retirement age of 67 years old [53].

e Terminal phase is the final 12 months of life; continuing phase is all the months between initial (first 12 months after diagnosis) and terminal phase.

f Medical cost includes hospital inpatient admissions, emergency room visits and outpatient services (includes chemotherapy, biologics, office visits, hospital visits).

g Expressed in terms of annual probability.

h Only to be applied to the first cycle.

Indirect costs related to patient work loss can be substantial [31]. To estimate this, time estimates associated with CRC care were used [32].

Incremental cost-effectiveness output was calculated by dividing the total incremental costs by the incremental effectiveness and reported as cost per QALY or cost per LYG. The adopted societal willingness-to-pay threshold was USD100,000 [33].

### Sensitivity Analyses

Cognizant of the many underlying assumptions and the limited randomized evidence base available, one-way sensitivity analyses, extensive one-way sensitivity analyses, and multivariable probabilistic sensitivity analyses (PSA) were performed to evaluate the impact of model assumptions on the study findings.

One-way sensitivity analyses, represented in the form of tornado diagrams, were conducted for the variables shown in Table 2. The incremental cost-effectiveness ratio (ICER) is contingent on the accuracy of estimates of these variables. The 95% confidence intervals from primary sources were used whenever such data were available; where absent, a ±20% range was applied with the exception of transition probabilities that were between 0 and 0.01. For these transition probabilities, the lower and upper bound limits of 0 and 10 times base case value (or 0.01, whichever greater) were applied respectively. For variables with values that varied during the simulated cycles, the ±20% range was calculated using the largest base case value. In addition, costs more than USD100,000 were varied widely from 50% to 200% of base case values to reflect the impact of outliers. Variables excluded from sensitivity analyses were starting age, background mortality, surveillance costs, and transition probability of non-fatal AE and fatal AE for the no treatment arm.

**Table 2:** Model Inputs for One-way Sensitivity Analyses.

	Input Parameter	Mean	Range tested
**Natural history** a **[16], [54]**	From ‘Remission with Unplanned Discontinued Treatment’ to ‘Recurrence’	Age-dependent	Not varied for analysis
	From ‘Remission with Unplanned Discontinued Treatment’ to ‘Death’	Age-dependent	Not varied for analysis
	From ‘Recurrence’ to ‘Death’	Age-dependent	Not varied for analysis
**Treatment effects**	**Relative risk of disease progression**		
**[9], [20]–[23], [42], [48]–[50], [55]**	Aspirin	0.53	0.33–0.86
	Capecitabine	0.78	0.67–0.91
	**Fatal adverse events** b		
	Aspirin	0.0008–0.0030	0–0.03
		(Age-dependent)	
	Capecitabine	0.003	0–0.03
	**Non-fatal adverse events** b		
	Aspirin	0.0036–0.0111	0–0.11
		(Age-dependent)	
	Capecitabine	0.0291	0.0233–0.0349
**Utilities** **[24], [25]**	Utility when on aspirin	0.999	0.7992–1.0
	Stage I: ‘Remission with Intervention’	0.84	0.5068–1.0
	Stage I: ‘Remission with Unplanned Discontinued Treatment’		
	Stage II: ‘Remission with Intervention’	0.86	0.5856–1.0
	Stage II: ‘Remission with Unplanned Discontinued Treatment’		
	‘Recurrence’	0.84	0.6048–1.0
**Costs in 2013 US**	**Drug Cost (Annual)** c		
**Dollars**	Aspirin	30	24–30
**[13], [27], [29], [31], [32], [35], [52], [56]**	Capecitabine	55,569	44,455–66,683
	**Indirect Cost (Patient’s Time)** d		
	‘Remission with Intervention’	255	204–306
	‘Remission with Discontinued Treatment’		
	‘Recurrence’	10,437	8,350–12,524
	**Medical Care Cost**		
	Cost of care for metastatic colorectal cancer	138,453	69,226–276,906
	**Adverse Events Cost**		
	Aspirin (fatal)	32,656–37,301	33,228–50,931
		(Dependent on age-dependent event rates)	
	Aspirin (non-fatal)	41,056–42,443	26,125–44,761
		(Dependent on age-dependent event rates)	
	Capecitabine (fatal)	53,820	43,056–64,583
	Capecitabine (non-fatal)	5,157	4,125–6,188

a Transition probabilities were derived from Stage I and II colorectal cancer patients (aged 65–69 when diagnosed) who were diagnosed between 1989 to 1993 to give 18–22 years of follow-up data. More details can be found in the File S1.

b For aspirin, the probabilities of fatal and non-fatal adverse events are applied only for the first five years; for capecitabine, probability of 0.0291 for non-fatal adverse events is applied only in the first year. An annual probability of 0 is applied for all other years.

c Cost of aspirin is applied only in the first five years; cost of capecitabine is applied only in the first year.

d The range for indirect cost is derived by multiplying the lower (−20%) or upper limits (+20%) of the time estimate by the respective applicable lower or upper limits of the median hourly wage.

Variables with high levels of uncertainty, identified as those with spread exceeding 50,000 from the initial one-way sensitivity analyses, were first subjected to extensive one-way sensitivity analyses to elucidate the robustness of the base case results before being included in the multivariable PSA. The ranges of values tested in the extensive one-way sensitivity analyses are 0 to 1 for all variables with this lower and upper bound limit, up to 10% for transition probabilities relating to fatal AE rates, up to 30% for transition probabilities relating to non-fatal AE, up to USD100,000 for drug cost, and up to USD600,000 for cost of metastatic CRC care. Multivariable PSA was conducted with a Monte Carlo simulation of 10,000 iterations using the appropriate distribution for the corresponding type of parameter (Table 3) [34]. Due to the limitations of the evidence available for the construction of the models in this study, a pragmatic approach to fitting distributions to parameters based on available information has to be taken [34]. Cost-effectiveness (CE) acceptability curves were then plotted with the percentage of cost-effective iterations against willingness-to-pay thresholds ranging from USD0 to USD100,000.

**Table 3:** Distributions of Model Inputs in the Probabilistic Sensitivity Analysis (PSA).

Input Parameter	Distribution	Mean^a^	Standard Deviation (SD)^a^
**Stage I**			
Cost of care for metastatic CRC in ‘Recurrence’ state	Gamma	138,453	630,923
Transition probability of fatal AE when on aspirin	Uniform	Age-dependent transition probabilities of fatal AE	
Transition probability of non-fatal AE when on aspirin	Uniform	Age-dependent transition probabilities of non-fatal AE	
Relative risk of disease progression when on aspirin	LogNormal	−0.635^b^	0.244^c^
Utility of taking aspirin	Beta	0.999	0.00383
Utility score of staying in ‘Remission with Intervention’	Beta	0.84	0.17
Utility score of staying in ‘Remission without Intervention’	Beta	0.84	0.17
**Stage II**			
Cost of 8 cycles of capecitabine	Gamma	55,569	1,329
Cost of care for metastatic CRC in ‘Recurrence’ state	Gamma	138,453	630,923
Relative risk of disease progression when on aspirin	LogNormal	−0.635^b^	0.244^c^
Relative risk of disease progression when on capecitabine	LogNormal	−0.248^b^	0.0781^c^
Transition probability of fatal AE when on aspirin	Uniform	Age-dependent transition probabilities of fatal AE	
Transition probability of fatal AE when on capecitabine	Beta	Alpha: 3	Beta: 990
Transition probability from non-fatal AE when on aspirin	Uniform	Age-dependent transition probabilities of non-fatal AE	
Utility of taking aspirin	Beta	0.999	0.00383
Utility score of staying in ‘Remission with Intervention’	Beta	0.86	0.14

a For parameters relating to relative risks, the figures have been rounded off to 3 significant figures for ease of reading.

b Expressed in u (mean in natural log).

c Expressed in sigma (standard deviation in natural log).

## Results

### Base Case Analysis

The results of the base case cost-effectiveness analysis are shown in Table 4. In both Stage I and II CRC, the base case analyses provide preliminary results to suggest that aspirin is a cost-effective option as compared to the other options.

**Table 4:** Base Case Cost-Effectiveness Analysis of Treatment Strategies.

	Stage I		Stage II		
	Aspirin	No treatment	Aspirin	Capecitabine	No treatment
**Costs (in 2013 US dollars)**					
Drug Cost	30	0	30	55,569	0
Surveillance Cost	590 to 1,927	590 to 1,927	590 to 1,927	590 to 2,785	590 to 1,927
Medical Care Cost	138,453	138,453	138,453	138,453	138,453
Indirect Cost (‘Remission’ state)	255	255	255	255	255
Indirect Cost (‘Recurrence’ state)	10,437	10,437	10,437	10,437	10,437
Adverse Event	32,656 to 42,443	0	32,656 to 42,443	5,157 to 53,820	0
**After 20 Cycles (without disutility of taking aspirin)**					
Total Cost	86,214	100,461	65,554	126,831	75,418
Cost differences	-	14,247	-	61,277^a^	9,864^a^
Total Quality-Adjusted Life Years (QALY)	9.57	9.37	8.66	8.42	8.51
QALY differences	-	-0.20	-	-0.24^a^	-0.15^a^
ICER (QALY)	-	Dominated	-	Dominated^a^	Dominated^a^
		(−72,500)		(−256,300)	(−63,700)
Total Life Years Gained (LYG)	11.39	11.16	10.09	9.81	9.91
LYG differences	-	−0.23	-	−0.28^a^	−0.18^a^
ICER (LYG)	-	Dominated	-	Dominated^a^	Dominated^a^
		(−60,900)		(−221,000)	(−55,400)
**After 20 Cycles (with disutility of taking aspirin** b **)**					
Total Cost	86,214	100,461	65,554	126,831	75,418
Cost differences	-	14,247	-	61,277^a^	9,864^a^
Total Quality-Adjusted Life Years (QALY)	9.56	9.37	8.66	8.42	8.51
QALY differences	-	−0.19	-	−0.24^a^	−0.15^a^
ICER (QALY)	-	Dominated	-	Dominated^a^	Dominated^a^
		(−73,900)		(−260,300)	(−65,300)
Total Life Years Gained (LYG)	11.38	11.16	10.08	9.81	9.91
LYG differences	-	−0.23	-	−0.27^a^	−0.17^a^
ICER (LYG)	-	Dominated	-	Dominated^a^	Dominated^a^
		(−62,100)		(−224,500)	(−56,800)

a Using the ‘Aspirin’ arm as the reference as it is the treatment with the lowest cost among all the comparison arms.

b A disutility of 0.001 (i.e. utility of 0.999) [25] was applied during the period aspirin was taken.

Note

• A treatment is dominated if it is both more costly and less effective than aspirin.

• The calculations of QALY and LYG reflected in the table have been rounded to the nearest 2 decimal places for ease of reading. As such, the summation some figures may vary slightly from the total figures reflected in the table. Similarly, the ICER results are reported to the nearest hundred.

The no treatment strategy remained dominated (i.e. more expensive and less effective than aspirin) in both stages. Similarly, capecitabine was also dominated. In general, although the differences in QALY and LYG of the dominated strategies were only 0.15 to 0.28 less than that of aspirin, the cost differences were substantial with a range of USD9,864 to USD61,277. The additional application of a utility of 0.999 during the period aspirin was taken did not appear to have an impact on the results.

### Sensitivity Analyses

#### One-way Sensitivity Analyses

**Figure 2:**
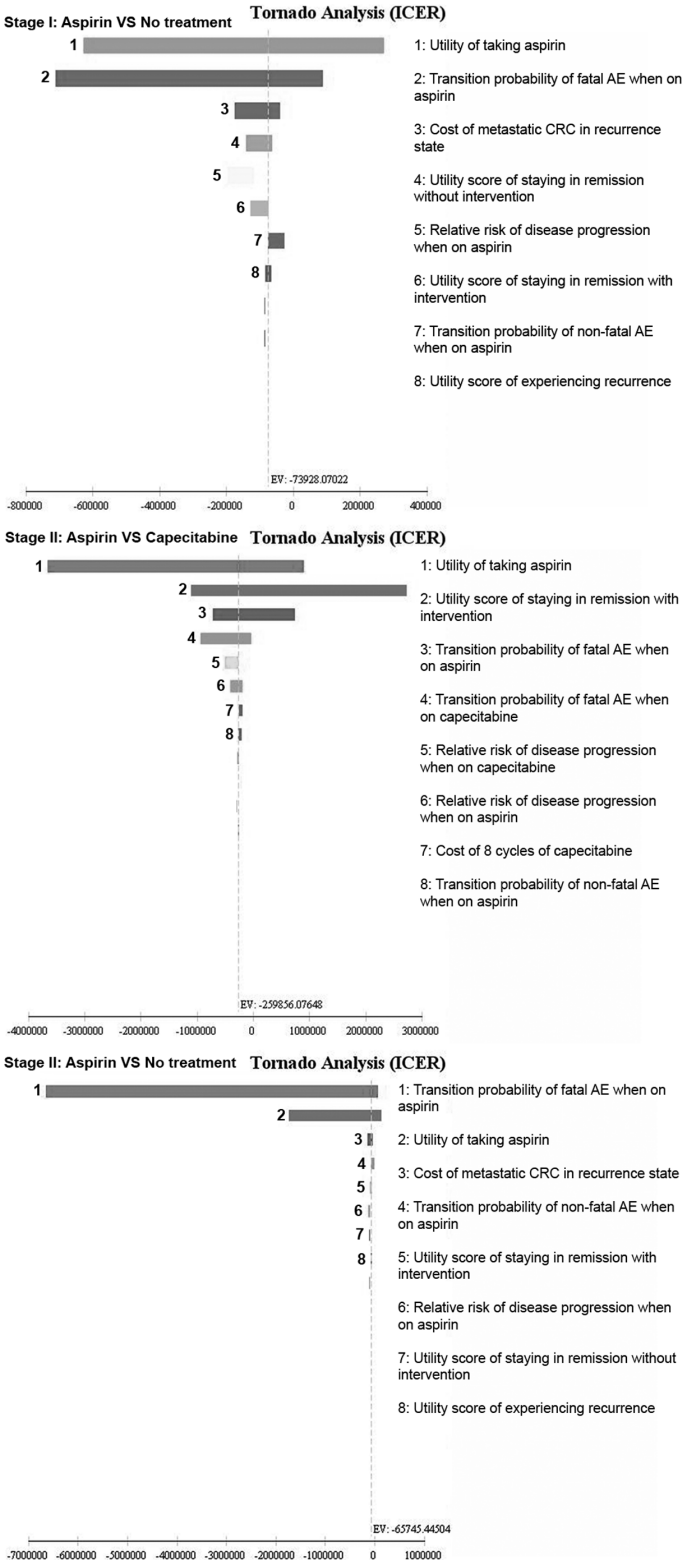
Tornado Analyses Diagrams of One-way Sensitivity Analyses. **AE**: Adverse event; **CRC**: Colorectal cancer.

Based on the one-way sensitivity analysis (Figure 2), the sensitive variables for Stage I CRC were: (i) utility of taking aspirin, (ii) transition probability of fatal AE when on aspirin, (iii) cost of metastatic CRC in recurrence state, (iv) utility score of staying in remission without intervention, (v) relative risk of disease progression when on aspirin, (vi) utility score of staying in remission with intervention, (vii) transition probability of non-fatal AE when on aspirin.

For Stage II CRC, the sensitive variables were: (i) utility of taking aspirin, (ii) utility score of staying in remission with intervention, (iii) transition probability of fatal AE when on aspirin, (iv) transition probability of fatal AE when on capecitabine, (v) relative risk of disease progression when on capecitabine, (vi) relative risk of disease progression when on aspirin, (vii) cost of 8 cycles of capecitabine, (viii) transition probability of non-fatal AE when on aspirin, and (ix) cost of metastatic CRC in recurrence state.

#### Extensive One-way Sensitivity Analysis

Overall, the extensive one-way sensitivity analysis (File S3) showed that the results of the base case were not affected to a large extent over wide ranges of the variables identified in the initial one-way sensitivity analyses. In Stage I CRC, aspirin was found to be cheaper and more effective (i.e. dominant) than the no treatment strategy when one of the following conditions occurred: (i) utility of taking aspirin was 0.948 or more, (ii) annual probability of fatal aspirin-related AE did not exceed 0.03%, (iii) cost of care for metastatic CRC was more than USD7,200, (iv) utility score of staying in ‘Remission without intervention’ was 0 to 1, (v) relative risk of disease progression when on aspirin was 0 to 0.96, (vi) utility score of staying in ‘Remission with intervention’ was 0 to 1, (vii) annual probability of non-fatal AE when on aspirin was 16.8% or less. Aspirin was dominated (i.e. more expensive and less effective) by the no treatment strategy when the relative risk of disease progression when on aspirin was 0.997 or more. The no treatment strategy could be a cost-effective alternative when the utility of taking aspirin was 0.909 or less, or when the annual probability of fatal aspirin AE was 0.57% or more.

For Stage II CRC, aspirin was the dominant option when: (i) utility of taking aspirin was 0.959 or more, (ii) utility score of staying in ‘Remission with intervention’ was 0.311 or more, (iii) annual probability of fatal aspirin AE was 0.31% or less, (iv) annual probability of fatal capecitabine AE was 10% or less, (v) relative risk of disease progression when on capecitabine was 0.507 or more, (vi) relative risk of disease progression when on aspirin was 0.959 or less, (vii) cost of 8 cycles of capecitabine was USD0 to USD100,000, (viii) annual probability of non-fatal aspirin AE was 12% or less, or (ix) cost of care of metastatic CRC in recurrence state was USD9,000 or more.

The no treatment strategy was a cost-effective alternative in Stage II when utility of taking aspirin was less than 0.931, annual probability of fatal aspirin AE was 0.49% or more, or relative risk of disease progression when on aspirin was 0.976 or more. Capecitabine could be a cost-effective option when the relative risk of disease progression when on capecitabine was less than 0.228.

#### Multivariable Probabilistic Sensitivity Analyses

**Figure 3:**
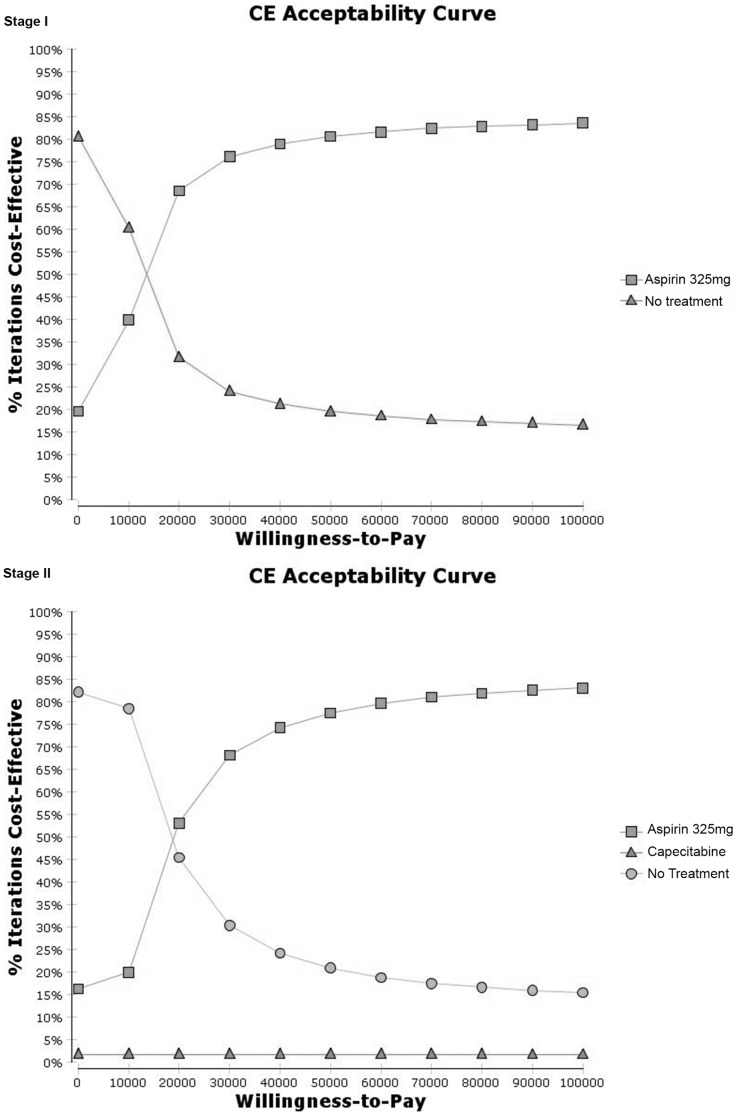
Cost-Effectiveness Acceptability Curves.

Using the assigned distributions of the variables identified to have high levels of uncertainty for each stage (Table 3), the CE acceptability curves generated using multivariable PSA are shown in Figure 3.

In Stage I CRC, aspirin was consistently cost-effective about 70% to 80% of the time as compared to the no treatment strategy when willingness-to-pay was varied from USD20,000 to USD100,000. At USD0, the no treatment strategy could be cost-effective 80% of the time. However, it decreased steeply to about 30% when the willingness-to-pay was USD20,000. For Stage II CRC, when willingness-to-pay was likewise varied, aspirin was cost-effective at least 50% of the time when the threshold laid between USD20,000 to USD100,000. Similarly, the no treatment strategy quickly became cost-effective at about 45% of the time at a threshold of USD20,000 despite being cost-effective for more than 80% of the time when the willingness-to-pay was USD0. Capecitabine could be cost-effective at about 2% of the time throughout the range of threshold tested.

## Discussion

Emerging evidence highlights some benefits of aspirin in several solid tumor cancers. In this first study of the hypothetical cost-effectiveness of aspirin in the adjuvant treatment of cancer, we found aspirin to be more cost-effective as compared to a no treatment strategy in Stage I and II CRC. Aspirin was also more cost-effective compared to capecitabine. Our PSA further showed aspirin to be cost-effective approximately 50% to 80% of the time in both stages when the willingness-to-pay threshold was between USD20,000 to USD100,000.

In our model, both no treatment and capecitabine were dominated by aspirin over wide ranges. However, no treatment or capecitabine (Stage II only) can be cost-effective alternatives in both stages if the utility of taking aspirin is below 0.909, aspirin’s annual fatal AE probability exceeds 0.57%, aspirin’s relative risk of disease progression is 0.997 or more, or when capecitabine’s relative risk of disease progression is less than 0.228.

Unlike capecitabine which has a well-defined regimen for use in Stage II CRC, there is a dearth of literature especially on the optimal dose and duration of aspirin therapy. In this study, we modeled aspirin to be a five-year therapy, covering the critical period where recurrence is most likely. Both aspirin and capecitabine were also assumed to exert their effects for the first five years of the simulation. Despite applying a utility of 0.999 for the period during which aspirin was taken in order to take into account the inconvenience of pill-taking, it did not bring about an appreciable difference. This is possibly due to the small margin of disutility assumed.

More recently, the cost of treatment of metastatic CRC has increased rapidly [35] with the incorporation of new biological treatments such as bevacizumab, cetuximab, panitumunab, aflibercept and regorafenib [36]–[38]. Thus an annual medical treatment cost of up to USD600,000 for metastatic CRC is no longer an obscure possibility. Additional analyses showed that aspirin remained cost-effective in both CRC stages even in an extreme scenario where the annual cost of care for recurrent metastatic CRC was USD6million.

We recognized that this study is not without limitations, mainly due to the uniform assumptions required. First, model inputs were estimated from a variety of sources. For example, indirect cost in the form of patient time was factored into cost inputs using certain wage and time estimates. These estimates did not include those incurred for AE and may not be generalizable to all CRC patients. Nevertheless, our findings in the sensitivity analyses remained similar over a wide range of estimates. Second, a number of the studies we drew data from, although consistent in their findings, were observational in nature [31], [32], [35], [39]. As such, limitations associated with observation studies (e.g. bias) would apply. Third, our model may be criticized for being overly simplified. However, given the paucity of data, a simpler model is probably more suited to the intended purpose of this study. In addition, our model did not permit individuals in the ‘Recurrence’ state to return to ‘Remission’ state even though this is clinically plausible [19]. However, as this happens only in a small number of CRC patients with liver metastases who could return to remission after surgical resection, it was not efficient to increase the complexity of the model to account for this low event probability.

In an attempt to give a conservative estimate of the cost-effectiveness of aspirin in CRC patients, potential cardio-protective and primary cancer prevention benefits of aspirin were not included. Although there is a recent study with a preliminary link of the use of aspirin to age-related macular degeneration [40], given the relatively rudimentary evidence and the small increase in risk, this effect was also not modeled. More recently, observational data has suggested that the tumor PIK3CA mutation or high tumor COX2 expression may serve as useful biomarkers for aspirin benefit [41]–[43]. Gene-expression analyses, although useful in prognosticating cancer relapses, have not yet been shown to predict adjuvant chemotherapy benefit. For these reasons, we have chosen to restrict our analysis to unselected CRC populations.

The National Cancer Institute has labeled aspirin’s activity in reducing CRC incidence and mortality as one of the most provocative questions in cancer [44], underscoring the importance and broad relevance of this treatment approach. Whereas *primary* cancer prevention with aspirin requires the treatment of large numbers of health individuals over prolonged periods of time, with toxicity and benefits finely balanced; aspirin’s ascendant role in the *secondary* prevention of resected cancers remains extremely attractive. Thus aspirin, if proven effective in prospective randomized trials is likely to play a unique role in the adjuvant treatment of Stage I and II cancers where large numbers of patients will need to be treated in order to prevent one cancer death. Our findings have two important implications. First, aspirin’s high cost-effectiveness in extremely low risk cancers alters the therapeutic paradigm of extremely low risks cancers and offers potential for adjuvant cancer treatment in a group of patients (i.e. Stage I CRC) that would currently undergo only observation. It supports a model of drug development away from traditional cytotoxics, where the risk of over-treatment is highest, towards repurposed old drugs such as aspirin. Second, the high cost-effectiveness of adjuvant aspirin underscores its broad social relevance to low income countries operating under constrained healthcare budgets. Lastly, the findings that aspirin is cost-effective even up to an extremely low therapeutic benefit ratio (i.e. a 1% relative risk reduction), draws attention to the difficulty in producing the requisite clinical evidence that is necessary to change clinical practice. A trial adequately powered for a hazard ratio of 0.99 in low risk cancer populations would require more than 300,000 subjects and would be impossibly expensive under existing development paradigms. Nonetheless, the potential benefits of aspirin as an adjuvant agent and its high cost-effectiveness justifies robust public support for research into its expanded use in the secondary prevention of cancer.

## Supporting Information

File S1 **Model Development and Validation.**
(DOCX)

File S2 **Transition Matrices of Stage I and II CRC.**
(DOCX)

File S3 **Model Input and Output of Extensive One-way Sensitivity Analyses.**
(DOCX)
