# Supplementary material for: Cost-Effectiveness of Aspirin Adjuvant Therapy in Early Stage Colorectal Cancer in Older Patients
Source: PLoS One. 2014 Sep 24;9(9):e107866. doi: 10.1371/journal.pone.0107866 (PMC4176715; doi:10.1371/journal.pone.0107866)
Supplement: File S1 — Model Development and Validation. (DOCX) [file pone.0107866.s001.docx]

**File S1: Model Development and Validation**

**Method**

Model-building

Model development of the no treatment arm (to mirror natural history) were performed using data derived from the Surveillance, Epidemiology, and End Results (SEER) Program SEER*Stat Database (version 8.1.2). The development of Stage I and II models were conducted separately using cases from the SEER 9 Regs Research Data, Nov 2012 Sub (1973 – 2010) <Katrina/Rita Population adjustment>. This database was chosen used as it provided the most recent 20-year data available in American Joint Committee on Cancer (AJCC) Third Edition staging with the widest possible geographical coverage.

Stage I CRC cases aged 65 to 69 at time of diagnosis with an initial diagnosis of cancer in the colon and rectum (based on ICD-O-3/WHO 2008 definitions) from 1989 to 1993 were extracted from the database. Between the respective years of initial diagnosis to 2010, the numbers of the following events occurring each year in the cohort were recorded: (i) diagnoses of first recurrences, (ii) deaths without recurrences, and (iii) deaths with recurrences. Events of each type were then summed up across the 5 years according to the number of years since initial diagnosis for use in the derivation of the annual transition probabilities for remission to recurrence, remission to death, and recurrence to death. This same process was repeated for Stage II CRC cases. Based on these annual transition probabilities, the models for Stage I and Stage CRC were constructed using TreeAge Pro 2013 (TreeAge Software, Inc. Williamstown, MA) to run over a 20-year period.

Internal Validation

Model outputs for each CRC stage were then compared with the SEER registry data used for model-building in terms of the following key events at the end of 20 simulated annual cycles: (i) number of subjects alive without recurrence, (ii) number of subjects alive with recurrence, and (iii) number of subjects dead. Chi-squared tests were then performed using R version 2.15.1 to compare the observed number of events from the registry with the predicted numbers from the models.

External Validation

20-year survival curves from the SEER 18 Regs Research Data + Hurricane Katrina Impacted Louisiana cases Nov 2012 Sub (1973-2010 varying) were separately extracted for Stage I and Stage II CRC cases 65 to 69 years of age at diagnosis and having an initial diagnosis from 1989 to 1993. Only data representing the observed survival which is an estimate of the probability of surviving all causes of death were used. These survival curves were then plotted on graphs and compared against the model outputs.

In a bid to further test for external validation using solely independent data, a second cohort of patients with similar age and CRC staging profile limited to those who had an initial CRC diagnosis for the period covering 1994 to 1998 were extracted from the SEER 9 Regs Research Data, Nov 2012 Sub (1973 – 2010) <Katrina/Rita Population adjustment> for comparison with model output in terms of number of key events. 17-year survival curves covering similar patient profile were also extracted from the SEER 18 Regs Research Data + Hurricane Katrina Impacted Louisiana cases Nov 2012 Sub (1973-2010 varying) for comparison with the model output.

**Results**

| Table S1. Total number of key events for observed and predicted data | | | | |
| --- | --- | --- | --- | --- |
|  | **Internal Validation**  **(over 20 years)** | | **External Validation**  **(over 17 years)** | |
|  | **SEER Registry** | **Model-derived** | **SEER Registry** | **Model-derived** |
|  | **Stage I** | | **Stage I** | |
| **Size of cohort** | 1,455 | 1,455 | 1,316 | 1,316 |
| **No. of subjects alive and in remission** | 431 | 431 | 633 | 480 |
| **No. of subjects alive and had recurrence** | 65 | 65 | 80 | 83 |
| **No. of subjects dead** | 959 | 959 | 603 | 752 |
| **p-value** | - | p = 1 |  | p < 0.001 |
|  | **Stage II** | | **Stage II** | |
| **Size of cohort** | 2,053 | 2,053 | 1,715 | 1,715 |
| **No. of subjects alive and in remission** | 500 | 500 | 642 | 517 |
| **No. of subjects alive and had recurrence** | 53 | 53 | 79 | 73 |
| **No. of subjects dead** | 1,500 | 1,500 | 994 | 1,125 |
| **p-value** | - | p = 1 |  | p < 0.001 |

| Figure S1-1. Comparison of predicted survival rates of Stage I and Stage II models against SEER registry’s observed survival curves (1989 to 1993) for external validation |
| --- |
| **Stage I** |
| % Surviving  Number of months |
| **Stage II** |
| % Surviving  Number of months |

**Observed all-cause survival (‘Observed’)** is an estimate of the probability of surviving all causes of death

| Figure S1-2. Comparison of predicted survival rates of Stage I and Stage II models against SEER registry’s observed survival curves (1994 to 1998) for external validation |
| --- |
| **Stage I** |
| % Surviving  Number of months |
| **Stage II** |
| % Surviving  Number of months |

**Observed all-cause survival (‘Observed’)** is an estimate of the probability of surviving all causes of death

The internal validation exercise found no statistically significant difference in the number of key events between the SEER database and the model output over a 20-year period.

For external validation, two cohorts of patients from two different time periods (1989 to 1993; 1994 to 1998) were used as the comparator to check on the survival profile of the model. In terms of the number of key events over a 17-year period (1994 to 1998), the Chi-squared results revealed statistically significant differences, with the number of subjects in the ‘Dead’ state for both Stage I and Stage II CRC being higher than the registry data. Nevertheless, in both time periods, the predicted survival rates were generally close to that observed in the respective SEER databases. A further comparison of the observed all-cause mortality using the 1989 to 1993 data with the models’ survival curves found that survival was similar although the predictions for Stage II had 3.9% to 7% higher survival than observed data in the first five years.
