# Supplementary material for: Cost-Effectiveness of Aspirin Adjuvant Therapy in Early Stage Colorectal Cancer in Older Patients
Source: PLoS One. 2014 Sep 24;9(9):e107866. doi: 10.1371/journal.pone.0107866 (PMC4176715; doi:10.1371/journal.pone.0107866)
Supplement: File S2 — Transition Matrices of Stage I and II CRC. (DOCX) [file pone.0107866.s002.docx]

**File S2: Transition Matrices of Stage I and II CRC**

| Table S2. Transition Matrices of Each Treatment Arm for Stages I and II | | | | | | |
| --- | --- | --- | --- | --- | --- | --- |
| **Stage I** | **No Treatment** | | | | | |
|  |  | **A** | **B** | **C** | **D** | **E** |
|  | **A** | 1-NFNoTx-tpC2D-BM-FNoTx | NFNoTx | 0 | tpC2D | BM+FNoTx |
|  | **B** | 0 | 0 | 1-BM | 0 | BM |
|  | **C** | 0 | 0 | 1-tpC2D-BM | tpC2D | BM |
|  | **D** | 0 | 0 | 0 | 1-tpD2E | tpD2E |
|  | **E** | 0 | 0 | 0 | 0 | 1 |
|  | **Aspirin** | | | | | |
|  |  | **A** | **B** | **C** | **D** | **E** |
|  | **A** | 1-NFNoTx-(tpC2D*RR1)-BM-FAsp | NFNoTx | 0 | tpC2D*RR1 | BM+FAsp |
|  | **B** | 0 | 0 | 1-BM | 0 | BM |
|  | **C** | 0 | 0 | 1-tpC2D-BM | tpC2D | BM |
|  | **D** | 0 | 0 | 0 | 1-tpD2E | tpD2E |
|  | **E** | 0 | 0 | 0 | 0 | 1 |
| **Stage II** | **No Treatment** | | | | | |
|  |  | **F** | **G** | **H** | **I** | **J** |
|  | **F** | 1-NFNoTx-tpH2I-BM-FNoTx | NFNoTx | 0 | tpH2I | BM+FNoTx |
|  | **G** | 0 | 0 | 1-BM | 0 | BM |
|  | **H** | 0 | 0 | 1- tpH2I-BM | tpH2I | BM |
|  | **I** | 0 | 0 | 0 | 1-tpI2J | tpI2J |
|  | **J** | 0 | 0 | 0 | 0 | 1 |
|  | **Aspirin** | | | | | |
|  |  | **F** | **G** | **H** | **I** | **J** |
|  | **F** | 1-NFAsp-(tpH2I*RR2)-BM-FAsp | NFAsp | 0 | tpH2I*RR2 | BM+FAsp |
|  | **G** | 0 | 0 | 1-BM | 0 | BM |
|  | **H** | 0 | 0 | 1-tpH2I-BM | tpH2I | BM |
|  | **I** | 0 | 0 | 0 | 1-tpI2J | tpI2J |
|  | **J** | 0 | 0 | 0 | 0 | 1 |
|  | **Capecitabine** | | | | | |
|  |  | **F** | **G** | **H** | **I** | **J** |
|  | **F** | 1-NFChemo-(tpH2I*RR3)-BM-FChemo | NFChemo | 0 | tpH2I*RR3 | BM+FChemo |
|  | **G** | 0 | 0 | 1-BM | 0 | BM |
|  | **H** | 0 | 0 | 1-tpH2I-BM | tpH2I | BM |
|  | **I** | 0 | 0 | 0 | 1-tpI2J | tpI2J |
|  | **J** | 0 | 0 | 0 | 0 | 1 |

Transition matrices above show the probability of transition from states in the rows to states in the columns.

Legend (in alphabetical order)

**BM**: Background mortality; **FAsp**: Transition probability of fatal adverse events when on aspirin; **FChemo**: Transition probability of fatal adverse events when on capecitabine; **FNoTx**: Transition probability of fatal adverse events when on no treatment (assumed zero); **NFAsp**: Transition probability of non-fatal adverse events when on aspirin; **NFChemo**: Transition probability of non-fatal adverse events when on capecitabine; **NFNoTx**: Transition probability of non-fatal adverse events for no treatment (assumed zero); **RR1**: Relative risk of disease progression when on aspirin for Stage I & II; **RR2**: Relative risk of disease progression when on aspirin for Stage I & II; **RR3**: Relative risk of disease progression when on capecitabine; **tpC2D**: Transition probability from ‘Remission with Discontinued Treatment’ to ‘Recurrence’ for Stage I; **tpD2E**: Transition probability from ‘Recurrence’ to ‘Death’ for Stage I; **tpH2I**: Transition probability from ‘Remission with Discontinued Treatment’ to ‘Recurrence’ for Stage II; **tpI2J**: Transition probability from ‘Recurrence’ to ‘Death’ for Stage II.

Stage I

**State A**: Remission with Intervention; **State B**: Treatment of Non-fatal Adverse Event; **State C**: Remission with Unplanned Discontinued Treatment; **State D**: Recurrence; **State E**: Death

Stage II

**State F**: Remission with Intervention; **State G**: Treatment of Non-fatal Adverse Event; **State H**: Remission with Unplanned Discontinued Treatment; **State I**: Recurrence; **State J**: Death
