# Supplementary material for: Cost-Effectiveness of Aspirin Adjuvant Therapy in Early Stage Colorectal Cancer in Older Patients
Source: PLoS One. 2014 Sep 24;9(9):e107866. doi: 10.1371/journal.pone.0107866 (PMC4176715; doi:10.1371/journal.pone.0107866)
Supplement: File S3 — Model Input and Output of Extensive One-way Sensitivity Analyses. (DOCX) [file pone.0107866.s003.docx]

| **File S3: Model Input and Output of Extensive One-way Sensitivity Analyses**   \| Figure S3-1. Model Input and Output of Extensive One-way Sensitivity Analyses in Stage I \| \| --- \| \| **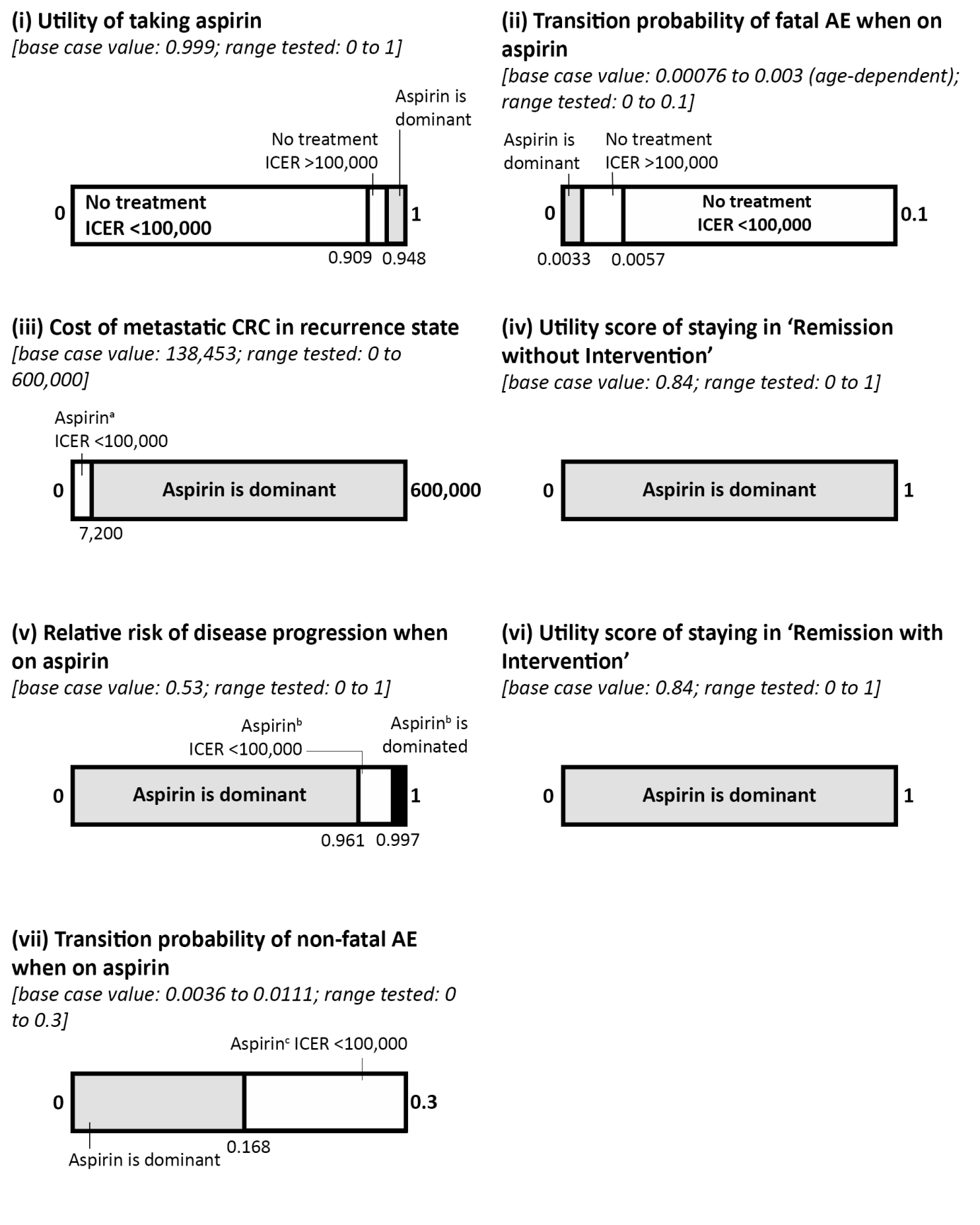** \| |
| --- | --- | --- |

^a^ No treatment strategy became the cheaper strategy, and hence, was the reference for comparison from 0 to 7,200.

^b^ No treatment strategy became the cheaper strategy, and hence, was the reference for comparison from 0.961 to 1.

^c^ No treatment strategy became the cheaper strategy , and hence, was the reference for comparison from 0.168 to 0.3.

Note

- A dominant strategy is one that is cheaper and more effective; a dominated strategy is more expensive and less effective.
- Unless otherwise specified, all strategies are compared against aspirin to give their respective incremental cost effectiveness ratio (ICER).
- Since the societal willingness-to-pay threshold adopted is USD100,000, an ICER below 100,000 is considered a cost-effective alternative; ICER exceeding 100,000 is not considered a cost-effective alternative.

| Figure S3-2. Model Input and Output of Extensive One-way Sensitivity Analyses in Stage II |
| --- |
| 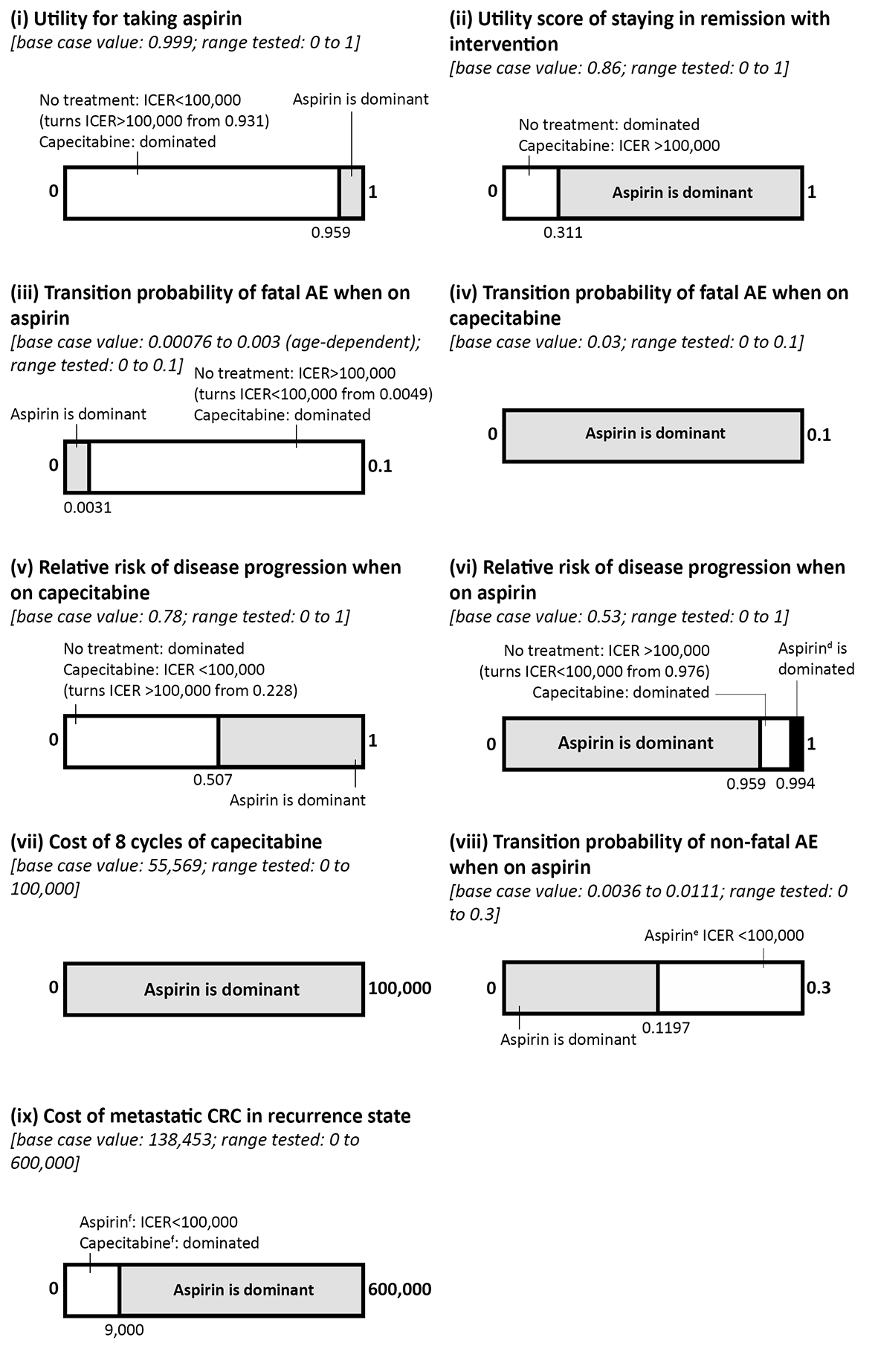 |

^d^ No treatment strategy became the cheapest strategy, and hence, was the reference for comparison from 0.994 to 1.

^e^ No treatment strategy became the cheapest strategy, and hence, was the reference for comparison from 0.1197 to 0.3.

^f^ No treatment strategy became the cheapest strategy, and hence, was the reference for comparison from 0 to 9,000.

Note

- A dominant strategy is one that is cheaper and more effective; a dominated strategy is more expensive and less effective.
- Unless otherwise specified, all strategies are compared against aspirin to give their respective incremental cost effectiveness ratio (ICER).
- Since the societal willingness-to-pay threshold adopted is USD100,000, an ICER below 100,000 is considered a cost-effective alternative; ICER exceeding 100,000 is not considered a cost-effective alternative.
